# Colonisation of a Phage Susceptible *Campylobacter jejuni* Population in Two Phage Positive Broiler Flocks

**DOI:** 10.1371/journal.pone.0094782

**Published:** 2014-04-14

**Authors:** Sophie Kittler, Samuel Fischer, Amir Abdulmawjood, Gerhard Glünder, Günter Klein

**Affiliations:** 1 Institute of Food Quality and Food Safety, University of Veterinary Medicine Hannover, Hannover, Germany; 2 Clinic for Poultry, University of Veterinary Medicine Hannover, Hannover, Germany; Charité-University Medicine Berlin, Germany

## Abstract

The pathogens *Campylobacter jejuni* and *Campylobacter coli* are commensals in the poultry intestine and campylobacteriosis is one of the most frequent foodborne diseases in developed and developing countries. Phages were identified to be effective in reducing intestinal *Campylobacter* load and this was evaluated, in the first field trials which were recently carried out. The aim of this study was to further investigate *Campylobacter* population dynamics during phage application on a commercial broiler farm. This study determines the superiority in colonisation of a *Campylobacter* type found in a field trial that was susceptible to phages in *in vitro* tests. The colonisation factors, i.e. motility and gamma glutamyl transferase activity, were increased in this type. The clustering in phylogenetic comparisons of MALDI-TOF spectra did not match the ST, biochemical phenotype and phage susceptibility. Occurrence of *Campylobacter jejuni* strains and phage susceptibility types with different colonisation potential seem to play a very important role in the success of phage therapy in commercial broiler houses. Thus, mechanisms of both, phage susceptibility and *Campylobacter* colonisation should be further investigated and considered when composing phage cocktails.

## Introduction

Phages are viruses that infect and kill bacteria. Even though they were widely used in the former Soviet Union, their use as antibacterial agents, has raised interest in the western world since antibiotic resistance has become a major problem in treating bacterial infections [1]. More recently, phages have been discussed for application in the food chain to control food- borne pathogens like *Campylobacter* [2]–[4]. *Campylobacter* is a Gram negative, motile bacterium and is the most frequent cause of zoonotic infections in developed countries with symptoms of mild diarrhoea, abdominal pain and fever. In 2011, there were 220,209 confirmed cases reported in the EU [5]. In about 1 out of 1000 cases, infections result in serious sequelae such as Guillain Barré Syndrome or Reactive Arthritis [6], [7]. *Campylobacter* is a commensal in birds, and broiler chickens are the main source for human infection [8], [9]. *Campylobacter jejuni* and *C. coli* are the most frequently isolated species causing human infection. Combating *Campylobacter* in primary production is considered to be effective for reducing human campylobacteriosis and different experiments have been carried out, confirming the efficacy of phages for reducing C*ampylobacter* load in chicken [10]–[14]. However, results have been highly variable in the onset and range of reduction and recently first field trials were performed to assess this issue under practical conditions. In two of three field trials a significant reduction was observed, but as in the other studies, the range of reduction varied widely and occurrence of different bacterial phenotypes may have influenced results of these studies [14].

Several studies have reported extensive biodiversity in *C. jejuni* isolates populating broiler chickens [15]–[18] and more than one strain can coexist in one broiler flock at a time [19]–[22]. Results indicate that phenotypic differences occur in closely related strains of *C. jejuni* and might be vital for the ability to colonise [23]. *C. jejuni* has a largely non- clonal population structure and generates genetic diversity through intra- and interspecies recombination [24]. In contrast to other intestinal pathogens, host factors instead of classical virulence factors seem to play a major role for pathogenesis of human campylobacteriosis [25]. Published studies to date, indicate that intestinal colonisation of chickens and humans is a multifactorial process [26]. The motility, imparted by the polar flagella, seems to play an important role in this process but studies indicate that it is influenced by many metabolic factors [26]–[29]. Additionally, motility is considered to be an important pathogenic determinant of *C. jejuni* [30]. Studies have demonstrated different expression patterns of flagella proteins between a poor and a robust coloniser strain in poultry [31], [32]. The genes of proteins involved in the modification of the flagellum are located in hyper variable regions of the *C. jejuni* genome [33]. While different studies found non- motile mutants exhibiting a decreased ability to enter cultural cells [34], [35] other studies have found a number of mutants with measurable defects in interaction with cultural cells that retained full motility [36]. It remains unclear how exactly motility and flagella structure are involved in colonisation of the chicken intestinal tract [30], [37], [38]. Tests on *Campylobacter* metabolism revealed wide variability and impact of amino acid catabolism on colonisation of the intestine [39]–[41], which is reasonable when considering that this bacterium uses intermediates of the tricarboxylic acid cycle as an energy source [25]. GGT is an enzyme involved in amino acid catabolism, providing utilisation of glutamine and glutathione and cellular protection against reactive oxygen species [42]. It is reported to be required for persistent colonisation [28]. The presence of the *ggt* gene was reported to correlate directly with expression of the GGT activity [28], [43].

While different studies have investigated the efficacy of applied phages, the development of resistance, and reduced susceptibility *in vivo* under a controlled environment [4], [10]–[13], [16], [44]–[49] little is known about the interaction of applied phages with different *C. jejuni* strains and phenotypes under commercial conditions. It is well known that a diversity of strains occurs in different flocks and slaughter groups [17]. This can result in generation of non- susceptible isolates [12], [32], [48] and the modulation of genes and proteins that have an effect on colonisation of susceptible strains [47], [50].

Interestingly, results of phage application trials using >3 week old birds by Loc-Carrillo et al. and Scott et al. [11], [51] suggested that phage resistant isolates can be generated by chromosomal inversion, but on the other hand express reduced ability to colonise the broiler intestine. However, Carvalho and colleagues who used 1 week old chicks found no differences in the ability of resistant and susceptible phenotypes to colonise the chicken gut [12]. It is worth highlighting that those differences may be associated with the difference in the age of the birds in the trials and the method used to sample the *Campylobacter* isolates from the birds post treatment.

The present study aims to investigate the bacterial population dynamics of different *C. jejuni* strains and phenotypes in two phage positive flocks from one farm. Furthermore, it concentrates in investigating two factors which are considered to be important for *Campylobacter* colonisation in chicken intestine: motility and gamma glutamyl transpeptidase (GGT).

## Material and Methods

### Field trials

The farm under investigation in this study was located in the north of Germany. It included 8 sheds in 2 buildings. Each building included two floors and two sheds on each floor. The sheds of the two broiler flocks under investigation were located on different floors of one building. Birds of both flocks were of the same age and housed on the same day. The field trial on this farm was originally part of a larger study by Kittler et al. [14]. Samples were taken from two phage positive flocks: Flock 1, which contained broilers experimentally treated with phage; and flock 2, which contained broilers that had been accidentally cross-contaminated with phage during the phage therapy trial. Application of phages in the field trials described by Kittler et al. [14] was performed according to German law. Formal approval of this study as an animal experiment was according to German law not necessary. However, the study was acknowledged by the Animal Welfare Committee of the University of Veterinary Medicine Hannover (competent ethics committee of the university). In accordance with German law permission to apply bacteriophages as feed additive was given by the competent authority (LAVES Az.: 41.3-63003-13/2011). Studies were performed on the farms R1 and M2. R1 was the farm under investigation in this study and is referred to throughout the manuscript as farm 1. Isolates of M2 (referred to throughout the manuscript as farm 2) were used for comparison of MLST analysis only. The owners gave their consent to participate in the study.

### Bacterial isolates

All 668 *C. jejuni* isolates used in this study were derived from farm 1, during the third phage application field trial described by Kittler et al. [14]. The field trial was carried out in 2012, testing the effect of a phage cocktail to reduce *Campylobacter* in commercial broiler chickens. Briefly, in each of two broiler chicken flocks 9 individual samples of faeces or caecal content were taken for *Campylobacter* spp. and phage enumeration. The flocks were naturally colonised by *Campylobacter*. Samples were taken when birds were 31, 32, 35 and 38 days old.

Depending on the availability of single colonies, up to 100 isolates from each flock and sampling time were collected. If the samples of a sampling time contained less than 100 colonies, all available single colonies were isolated. This altogether resulted in 668 collected isolates from a total of 55 *Campylobacter* positive samples. Only 15 isolates from flock 1 at day 31 were isolated and >65 isolates for all other samplings. The isolates were stored in skimmed milk as described below. For further examination, isolates were grown in Preston selective broth (Oxoid, Germany) for 48 h under microaerobic conditions and subsequently cultivated on Karmali agar plates (Oxoid, Germany).

### Entry of phages

As described in detail by Kittler et al. [14] phages were applied to flock 1 via drinking water after sampling of faeces for *Campylobacter* and phage detection from 31 day old birds. The phage cocktail consisted of 4 lytic, well characterised phages of the British phage typing scheme (NCTC 12672, 12673, 12674 and 12678). Flock 2 did not receive any phages. However, in flock 2 cross contamination occurred. Phages were found in the samples of this flock four days after phages had been applied in flock 1 [14].

### Detection of phages and *Campylobacter*

All samples were investigated for presence of phages and *Campylobacter* as previously described [14]. Presumptive *Campylobacter* colonies were picked from two plates containing <100 single colonies. They were cultivated on Karmali agar plates for 24 h and stored in skimmed milk at −80°C. Afterwards, they were examined for motility and typical cell morphology under the microscope and for positive catalase and oxidase reaction before storage.

### Multi Locus Sequence Typing

A total of 18 selected isolates from the two flocks were characterised by MLST analysis according to the procedure outlined by Dingle et al. [52]. The amplification and sequencing primers were obtained from *C. jejuni* PubMLST webpage. The seven housekeeping gene loci *aspA* (aspartase), *glnA* (glutamine synthetase), *gltA* (citrate synthase), *glyA* (serine hydroxyl methyl transferase), *pgm* (phosphor glucomutase), *tkt* (transketolase) and *uncA* (ATP synthase alpha subunit) were used. Sequence files were read, assembled, evaluated, aligned and compared to the reference set of alleles using BioNumerics 7.1 software (Applied Maths, Belgium). The 18 selected isolates were phylogenetically compared to 7 isolates of two other, similarly performed field trials [14] and to 314 strains isolated by members of the FBI-Zoo research network from different sources in Germany 2007–2011 [23]. Table 1 summarises the isolates and applied methods.

**Table 1:** Isolates used for MLST and MLST data analysis, phage susceptibility GGT activity test and motility assay.

					Method				
Reference	Source		Number of Isolates	Species	GGT test	MLST^a^	MLST data analysis	phage susceptibility	motility assay
Phage application field trials by Kittler et al.^b^			675						
	farm1	chickens	18	*C. jejuni*	+	+	+	+	+
		chickens	650	*C. jejuni*	+	/	/	+	+
	farm2	chickens	7	*C. jejuni*	/	+	+	/	/
PubMLST, Oxford, UK, Gripp et al.^c^			314						
		animal	89	*C. jejuni*	/	/	+	/	/
		food	93	*C. jejuni*	/	/	+	/	/
		human	131	*C. jejuni*	/	/	+	/	/
		other	1	*C. jejuni*	/	/	+	/	/

/ Method not applied.

+ Method applied in this study.

a including isolates from Kittler et al 2013 (18 isolates from farm 1, 7 isolates from farm 2).

b Reference number [14].

c Reference number [23].

### GGT activity of *Campylobacter* isolates

All 668 isolates were tested for GGT activity by using Gamma- Glutamyl- Aminopeptidase Diatabs (Rosco Diagnostica, Taastrup, Denmark) according to the manufacturer's instructions. The test was performed in 48 well microtiter plates. Positive- and negative- controls were prepared for each assay using the well characterised strains NCTC 12662 and 19660- 10 from our laboratory reference stock.

### Phage susceptibility of *Campylobacter* isolates

Susceptibility of *Campylobacter* isolates to the phages in our cocktail was tested, using a modification of the agar overlay method previously described [53]. Briefly, 100 µl phage test suspension and 100 µl of the respective *Campylobacter* isolate (McFarland 3, equating log_10_ 7–9 cfu/ml data not shown,in 10 mmol MgSO_4_) were added to 5 ml molten NZCYM Overlay agar (0.7% agar agar) and poured on plates of NZCYM base agar (1.5% agar agar). For preparing McFarland 3, *Campylobacter* was grown on MH blood plates (Oxoid, Germany) for 17 hours. The phage test suspension harboured all four cocktail phages in equal parts at an overall concentration of log_10_ 4 pfu/ml.

Plates were incubated for 24 h and isolates were ranked in four susceptibility classes: Non- susceptible isolates without plaque formation; low susceptibility isolates (1–10 pfu/ 0.1 ml), medium susceptibility isolates (10–100 pfu/ 0.1 ml), high susceptibility isolates (100–1000 plaques/ 0.1 ml). *C. jejuni* NCTC 12662 was used for positive (log_10_ 4 pfu/ml test suspension) and negative control (no test suspension).

### Motility assay of *Campylobacter* isolates

Motility of all 668 isolates was tested by a method described previously by Gaynor et al. [34] with slight modification. Briefly, 1 µl McFarland 3 suspension of the isolate was stabbed into NZCYM agar plates containing 0.7% agar (Oxoid, Germany). McFarland 3 was prepared as stated above using cultures that had been incubated for 17 h. After 24 h incubation, the diameter of the colonised zone was measured.

### MALDI-TOF analysis

41 Representative isolates of all samplings and phenotypic groups were analysed by matrix-assisted laser desorption ionisation-time of flight mass spectrometry (MALDI-TOF MS).

The procedure was adapted from the ‘ethanol/formic acid extraction’ validated by the manufacturer (Bruker Daltonics GmbH, Germany). Briefly, isolates were cultivated for 17 h and 3–4 colonies were suspended in tubes containing 150 µl bidistilled water and 450 µl absolute ethanol. Subsequently they were stored at −20°C. After thawing they were centrifuged at 2000×g, the liquid phase was removed and the pellet was resuspended in 25 µl formic acid (70%), mixed with 50 µl acetonitrile and was centrifuged. 1 µl of the supernatant was placed onto a polished steel MALDI target plate (Bruker Daltonik GmbH, Germany) at four MALDI target positions per strain. After drying at room temperature each sample was overlaid with 1 µl of matrix containing α-cyano-hydroxy-cinnamic acid in 2.5% trifluoroacetic acid and 50% acetonitrile in water (Bruker Daltonik GmbH). They were dried at room temperature and the MALDI-TOF MS measurement was performed using the Bruker Biotyper (Bruker Daltronik GmbH). Raw spectrum data were analysed using Bionumerics Software 7.1.

### Biochemical phenotyping

The API Campy test system (Biomerieux, France) was used for biochemical phenotyping of >250 representative isolates of all groups and samplings according to the manufacturer's instructions. Briefly, an overnight culture of each isolate was prepared and suspended in NaCl and AUX medium. Subsequently, microtubes of the API Campy strip were charged and incubated. The reactions were read according to the manufacturer's instructions and analysed using Bionumerics Software 7.1.

### Data analysis

SAS 9.3 software was used for statistical analysis. For detecting significant differences in *Campylobacter* counts, the Wilcoxon rank sum test was used.

For evaluating susceptibility distribution and GGT activity the Chi-square test or Fisher-Yates-test were used and statistical significant differences in motility were detected using the t-test or Wilcoxon-rank sum-test.

MLST profiles, MALDI-TOF spectra and Biochemical profiles of isolates were compared by using the Minimum Spanning Tree plugin, and the cluster analysis tool of the BioNumerics 7.1 software.

## Results

A total of 668 *C. jejuni* isolates were collected from two flocks before and after *Campylobacter*-phage application on a commercial broiler farm. The isolates were analysed for phage susceptibility, motility and biochemical phenotype. For phylogenetic comparison of selected isolates MLST sequence type (ST) and clonal complex (CC) analysis was carried out.

**Figure 1:**
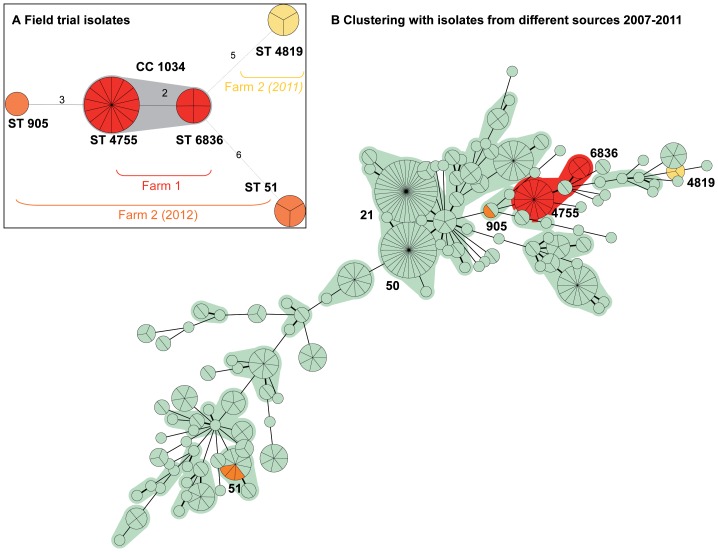
Minimum spanning tree of MLST allelic profiles depicting the clustering of the sequence types. Red dots: Isolates presented in this paper (field trial farm 1*) Isolates for comparison: Isolates of farm 2* from 2011 (yellow dots) and 2012 (orange dots), isolates published by Gripp et al. 2011 (green dots) Clonal complexes (maximum two allele difference between neighbouring STs) are indicated by coloured shading around circles. Most important STs and CCs are indicated by numbers. *including isolates from Kittler et al. 2013 (18 farm 1 isolates, 7 farm 2 isolates).

MLST analyses of 18 selected isolates (9 isolates from each flock of the investigated farm 1, thus including isolates of all samplings) revealed two STs that differed in two of seven MLST loci: 14 isolates belonged to ST 4755, whereas a new ST 6836 was identified for 4 isolates from flock 1 (Table 2). The new ST differed from ST 4755 in the sequence of *aspA* and *pgm* housekeeping genes. Both STs were related, belonging to ST-1034 CC (Fig. 1, farm 1: red isolates). These 18 isolates from farm 1 were compared to 7 isolates from other phage application field trials on a different farm (farm 2) in 2011 and 2012 (Fig. 1, farm 2: yellow and orange isolates) [14]. Results are shown in Fig. 1 A. The STs differed in at least 3 allels. Interestingly, the STs of the 7 isolates from the other field trials were not related to each other and differed in a minimum of 5 allels. The islolates analysed by Gripp et al., deriving from different human, animal, food and environmental sources in Germany from 2007–2011, were used for comparison because they were well characterised and tested for a set of metabolic properties [23]. These properties included metabolic variation, energy harvest and genes proposed to be involved in pathogenesis and metabolism such as motility and presence of the *ggt* gene. Results of comparing MLST profiles of field trial isolates and the isolates analysed by Gripp et al. are shown in Fig 1 B. The two STs of the investigated farm 1 isolates showed a minimal difference in their allelic profile compared to the ST 1709 isolates analysed by Gripp et al. [23]. The ST 1709 isolates derived from poultry meat in 2008 (Isolate FBI 04313; pubmlst.org) and from a laying hen 2009 (Isolate FBI 04343; pubmlst.org) [23].

**Table 2:** GGT activity, MLST (ST), clonal complex (CC) and phage susceptibility of representative Isolates of the investigated field trial^a^.

	Isolate	Species	GGT activity	ST	CC	Phage susceptibility^b^
**Flock 1**	3–554	*C.jejuni*	−	6836	ST-1034 complex	high
	3–102	*C.jejuni*	−	6836	ST-1034 complex	none
	3–827	*C.jejuni*	−	6836	ST-1034 complex	none
	3–381	*C.jejuni*	+	6836	ST-1034 complex	none
	3–353	*C.jejuni*	−	4755	ST-1034 complex	high
	3–310	*C.jejuni*	+	4755	ST-1034 complex	high
	3–840	*C.jejuni*	+	4755	ST-1034 complex	high
	3–891	*C.jejuni*	+	4755	ST-1034 complex	none
	3–540	*C.jejuni*	+	4755	ST-1034 complex	none
**Flock 2**	3–003	*C.jejuni*	−	4755	ST-1034 complex	high
	3–013	*C.jejuni*	−	4755	ST-1034 complex	high
	3–058	*C.jejuni*	−	4755	ST-1034 complex	none
	3–722	*C.jejuni*	−	4755	ST-1034 complex	none
	3–005	*C.jejuni*	+	4755	ST-1034 complex	high
	3–714	*C.jejuni*	+	4755	ST-1034 complex	high
	3–791	*C.jejuni*	+	4755	ST-1034 complex	high
	3–032	*C.jejuni*	+	4755	ST-1034 complex	none
	3–721	*C.jejuni*	+	4755	ST-1034 complex	none

a Farm 1.

b Phage susceptibility was classified in four classes: High, medium, low and no phage susceptibility. Each class represents ten- fold reduction compared to the superior class.

The 668 isolates were further examined. Two phenotypes differing in the presence or absence of GGT activity were identified. Results of *Campylobacter* and phage detection in samples and frequency of GGT activity in both flocks are shown in Table 3. The isolates with GGT activity were significantly more often susceptible to phage infection than isolates displaying no GGT activity (p<0.0001, N = 668, Table 4). However, as displayed in Table 4, 58 isolates did not express these most frequent phenotypic combinations of GGT activity and phage susceptibility (Table 4, no GGT activity and phage susceptibility or GGT activity and no phage susceptibility). These isolates originated from all samplings of the field trial.

**Table 3:** Phages and *Campylobacter* isolated on farm 1 and distribution of gamma glutamyl transferase activity.

		positive samples/No. of samples			
	Age of birds (days)	Phages	*Campylobacter*	Number of isolates without GGT activity (%)	Number of isolates with GGT activity (%)
**Flock 1**	31	0/9	1/9	15 (100)	0 (0)
	32	2/9	7/9	96 (96)	4 (4)
	35	8/9	7/9	40 (40)	59 (60)
	38	8/9	9/9	9 (13)	58 (87)
**Flock 2**	31	0/9	5/9	5 (5)	91 (95)
	32	0/9	8/9	6 (6)	92 (94)
	35	8/9	9/9	0 (0)	99 (100)
	38	7/9	9/9	15 (16)	79 (84)

A significant decrease of log_10_ 1.8 cfu/g in mean *Campylobacter* counts took place in the investigated flock 2 from day 35 to day 38 (p = 0.00078).

All samples were faecal samples except for the last sampling in the flocks which were caecal samples from the slaughter- house. Detection limit was 50 cfu or pfu/g, respectively for phages and *Campylobacter* in all samples.

**Table 4:** Susceptibility of *Campylobacter jejuni* isolates with and without gamma glutamyl transferase (GGT) activity on farm 1.

		No GGT activity	GGT activity
**Non- susceptible**		**161** a	**33** a
**Susceptible**	low	1	23
	medium	8	38
	high	16	388
	**total**	**25** a	**449** a

a Chi-square test p<0.0001.

**In flock 1** a phage cocktail was applied when birds were 31 days old [14]. The results displayed in Table 3 indicate that phages spread rapidly in the flock until they were found in 8 out of 9 samples after four days. Results of *Campylobacter* detection during the same period (Table 3) showed one positive sample (detection limit 50 cfu/g) when birds were 31 days old before phage application. Results of subsequent testings indicated a rapid spread of *Campylobacter* until all samples harboured *Campylobacter* after 10 days, suggesting colonisation occurring despite the presence of phages (Table 3; Flock 1).

**Figure 2:**
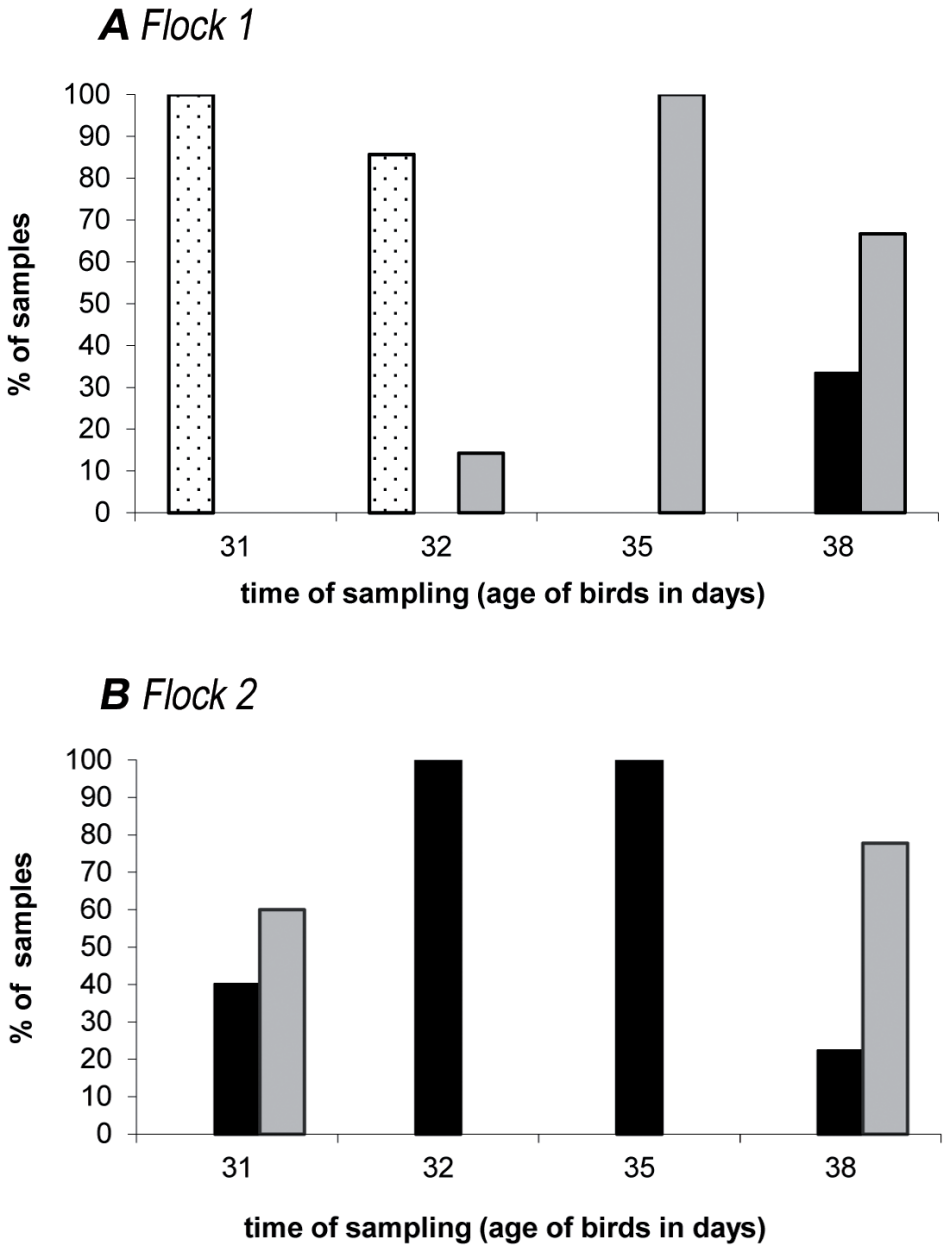
Phage susceptible *Campylobacter jejuni* isolates colonised both flocks of the investigated field trial (farm 1). White bars: Samples containing non- susceptible isolates only Grey bars: Samples containing susceptible and non- susceptible isolates Black bars: Samples containing susceptible isolates only For each sampling time n = 9 samples and >65 isolates (n = 15 for flock 1 at day 31).

Distribution of GGT activity among isolates at different times is shown in Table 3. Isolates with GGT activity were isolated mainly after phage application and results indicate a rapid spread and improved colonisation compared to isolates without GGT activity (Table 3; Flock 1). Looking at Fig. 2; A, non- susceptible isolates only were isolated from most samples on the first two samplings, changing over to both, susceptible and non- susceptible isolates, in all samples at the third sampling. Thus, results indicated that the susceptible isolates spread rapidly while non- susceptible isolates declined (Fig. 2; A). As displayed in Table 2, neither GGT activity nor phage susceptibility coincided with a certain MLST profile.

**In flock 2**, entry of phages occurred due to unintended cross- contamination and horizontal transmission, in contrast to phage application in flock 1 [14]. While no phages were detected from fecal samples tested at day 32, three days later 8 out of 9 samples were tested positively (Table 3; Flock 2). More than 50% of the samples tested positive for *Campylobacter* when birds were 31 days old and subsequent spreading was observed until all samples were *Campylobacter* positive 4 days later (detection limit 50 cfu/g).

Isolates with and without GGT activity were present at day 31 in flock 2 but 100% of tested isolates showed GGT activity four days later. However, at the last sampling, isolates without GGT activity reappeared when numbers of *Campylobacter* were reduced (Table 3; Flock 1). In this flock most samples harboured both susceptible and non- susceptible isolates at the beginning of observation (Fig. 2; B), but only susceptible isolates were isolated one day later. However, non- susceptible isolates reappeared six days later, at the last sampling (Fig. 2; B).

**Figure 3:**
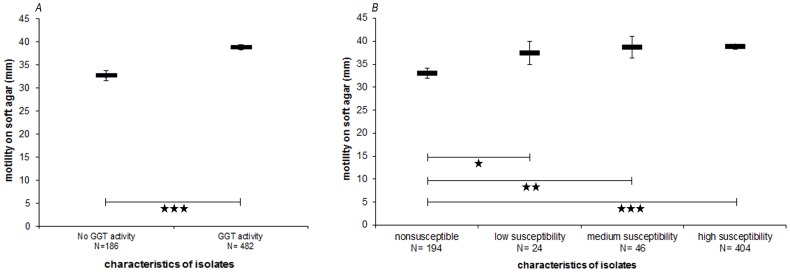
Mean motility of *C. jejuni* differed significantly between isolates with diverging GGT activity and susceptibility. Motility was tested on semisolid agar inoculating drops of 1 µl McFarland 3 (approx. log_10_ 8 cfu/ml) suspension. Differences of p<0.05 were considered as significant. Stars on vertical lines represent level of significance (***:P<0.0001; **:P<0.001; *:P<0.05). Error bars represent standard error of the mean. (A) Comparison of mean motility in isolates with and without GGT activity (B) Comparison of mean motility of isolates exibiting different phage susceptibility classes.

To determine if alteration of susceptibility or GGT activity correlated with changes in motility of the isolates, a motility assay was performed. Results are shown in Figure 3. Isolates with GGT activity showed a significantly higher motility than isolates without GGT activity (p<0.0001). Isolates were ranked in four susceptibility classes and even the lowest susceptibility class with 100 fold reduced susceptibility (1–10 plaques compared to 100–1000 in fully susceptible control) expressed a significantly higher motility compared to the non- susceptible isolates (Fig. 3 B; p = 0.0288 for low susceptibility; p = 0.0060 for medium susceptibility; p<0.0001 for high susceptibility).

## Discussion

In this study the dynamics of *Campylobacter* colonisation in presence of phages in two broiler flocks was investigated. MLST, MALDI-TOF and biochemical analysis, phage susceptibility and motility as well as GGT activity were examined.

The results of the present study indicate that a phage susceptible *C. jejuni* subpopulation with increased motility and GGT activity could overgrow a non- susceptible subpopulation in the presence of phages. Susceptibility of the isolates was only tested *in vitro*. Even though we used standard test methods for phage susceptibility, we cannot rule out that differences between *in vitro* and *in vivo* susceptibility existed. A reduced ability of phages to kill *in-vitro*-susceptible *Campylobacter in vivo* was reported in another study [11]. Nevertheless, phage replication occurred during the trials and is a sign of sufficient concentrations of susceptible *Campylobacter*. According to pharmacokinetic experimental and modelling approaches of Cairns et al. [46] minimum concentrations of approx. log_10_ 4 cfu/g of non-culturable *Campylobacter* isolates must have been present, if phages did not replicate in the susceptible *Campylobacter* that were isolated. Reappearance of non- susceptible isolates in flock 2 occurred when phages reached high concentrations and *Campylobacter* counts were reduced in this flock [14]. Even though no significant *Campylobacter* reduction occurred in flock 1, phage replication up to a mean of log_10_ 5 pfu/g caecal content was observed [14]. These findings suggest that *Campylobacter* may have been under selective pressure of phage induced lysis in this trial. These results support the findings of Loc Carrillo et al. and Scott et al. [11], [47], [51] who found that a resistant phenotype had fitness costs and was rapidly dominated by a susceptible phenotype *in vivo*. Scott et al. [47] found dominance of the susceptible strain only when phages were absent, which is in contrast to our findings (Table 3). However, Scott et al. [47] found a horizontal gene transfer of >112 kb to be responsible for change of MLST ST and loss of susceptibility while the metabolic features of the dominant strain remained unclear. Nonetheless, *in vivo* experiments might not reflect conditions in commercial broiler houses.

MLST analysis of the 18 selected isolates found presence of STs 4755 and 6836. Both STs belonged to the ST-1034 CC. They differ in the loci *aspA* and *pgm* and are closely related to each other. Previously published *Campylobacter* isolates from poultry sources were found to differ in only one allele compared to each of these STs [23], demonstrating that the isolates of the investigated field trail were closely related to different isolates from other poultry sources. Isolates from other field trials revealed unrelated STs (Fig. 1). This finding is in accordance with Bull et al. [54] who found unrelated STs being present in one flock. Different susceptibility classes and GGT activity were found for isolates having the same ST (Table 2). These results are in contrast to Scott et al. [47] who found resistance to be associated with change of MLST locus *pgm* allelic profile. All 668 isolates were tested for phage susceptibility and were subdivided into four levels of susceptibility: Non- susceptible, low susceptible, medium susceptible and high susceptible correspondent to the number of observed plaques (10 and 100 fold reduced for medium and low susceptibility compared to control NCTC 12662). In both flocks an increase of phage susceptible isolates was observed during the trial. When numbers of *Campylobacter* were reduced in flock 2 [14], non- susceptible isolates reappeared (Fig. 2). GGT activity was found to be correlated with phage susceptibility (p<0.0001, Table 4). Due to the fact that some isolates displayed phage susceptibility but no GGT activity or vice versa (Table 4), results indicate that there is no direct link between susceptibility and GGT activity. It is more likely that the GGT active phenotype is linked to other mechanisms which determine phage resistance. We explicitly looked for new STs among the isolates that displayed unusual GGT- phage susceptibility phenotypes (Table 4). These isolates are thus overrepresented in Table 2. However, they displayed the same STs as the frequent phenotypes did. As they occurred in low numbers in all samplings, they do not seem to play a major role in the population dynamics of this field trial. In order to detect additional strains or subtype- clusters we looked for clustering of isolates using BioNumerics 7.1 analysis for MALDI-TOF raw spectra and ApiCampy phenotypes. Isolates from all samplings and all possible GGT- phage susceptibility phenotypes were included in these tests. The typing methods applied were performed consecutively, i.e. when MLST and ApiCampy did not reveal enough detail, MALDI-TOF was additionally applied. Thus the isolates selected for each test varied slightly. Clustering by ApiCampy was not reliable as was indicated by branch quality error flags (data not shown). The cluster analysis of raw spectra using peak based pearsson correlation indicated two reliable clusters (Fig. S1). However, they included different STs and all combinations of GGT-phage susceptibility phenotypes (Fig. S1). These results suggest that no additional strain or subtype was isolated. In recent studies GGT was found to be important for the colonisation of chickens and was associated with severe human cases [28], [55], [56]. To investigate further reasons for the superiority in colonisation of the phage susceptible GGT positive phenotype, we investigated motility of all isolates. Motility is considered to be important for *Campylobacter* colonisation [25], [27]. An increased motility of the susceptible phenotype was found (Fig. 3), which could explain why susceptible *C. jejuni* isolates replaced non- susceptible isolates in the presence of phages, since GGT activity and increased motility might promote colonisation potential of this predominant phenotype. Thus, the results of previous *in vivo* studies were confirmed which found reduced motility of phage resistant phenotypes [51], [57] and sensitive revertants overgrowing the resistant phenotype [47]. In contrast, Carvalho at al. [12] found no differences in colonisation potential of resistant and susceptible isolates.

Different mechanisms have been reported that influence phage susceptibility of *Campylobacter*. These mechanisms can cause reversible or non-reversible genetic or metabolic changes and can thus lead to alteration in genotype or phenotype [16], [47], [48], [50], [51], [57], [58]. While some of these alterations might be reflected in MLST allelic profile, others might not [55], [59]. MLST STs and phage susceptibility did not coincide in our trial, thus demonstrating that the mechanism responsible for change of the MLST housekeeping genes *pgm* and *AspA* did not influence susceptibility of the isolates. In contrast, the presence of susceptible and non- susceptible isolates of both STs implies that other, very variable mechanisms might be responsible for the change of susceptibility and correlation with motility and GGT activity. Possible interpretations of this phenomenon include phase variation and pleiotropic effects as reported for other phage- resistant bacteria [60]–[63]. Structural features such as the flagellum and CPS are known to undergo phase variation [50], [64] and might contain receptor molecules necessary for phage adsorption [65]. Thus, in our study phase variation might have occurred and might have had pleiotropic effects on GGT activity. Furthermore, two separate mechanisms might have mediated resistance to different cocktail phages and have had effects on different metabolic features of the isolates. Further research is needed for a better understanding of underlying mechanisms.

Despite successful colonisation of the susceptible phenotype, Kittler et al. [14] demonstrated reduction of *Campylobacter* concentration in chicken caeca of the second flock. Reduced numbers of *Campylobacter* in phage positive flocks were also reported previously by Atterbury et al. [45] in an epidemiological investigation.

## Supporting Information

Figure S1 **Dendrogram of MALDI-TOF mass spectra and MLST sequence and phenotypes of** ***Campylobacter jejuni*** **isolates.** Sequence and phenotypes of isolates are not consistent with clustering of mass spectra. The dendrogram was generated using peak based pearsson correlation and UPGMA algorithm in BioNumerics 7.1. Error flags indicate branch quality by standard deviation associated with each cluster. Error flags which don't overlap indicate consistent clustering (green error flags). While overlapping error flags indicate non-consistent clusters (red error flags). Triangles display GGT activity (white triangle: no GGT activity, black triangle: GGT activity). Hexagons display phage susceptibility (white hexagon: non susceptible, black hexagon: susceptible). * Test result with low sensitivity: no distinct GGT phenotype. n.d. ST of this isolate was not tested.
(TIF)
