# Supplementary figures and images for: Colonisation of a Phage Susceptible Campylobacter jejuni Population in Two Phage Positive Broiler Flocks
Source: PLoS One. 2014 Apr 14;9(4):e94782. doi: 10.1371/journal.pone.0094782 (PMC3986380; doi:10.1371/journal.pone.0094782)

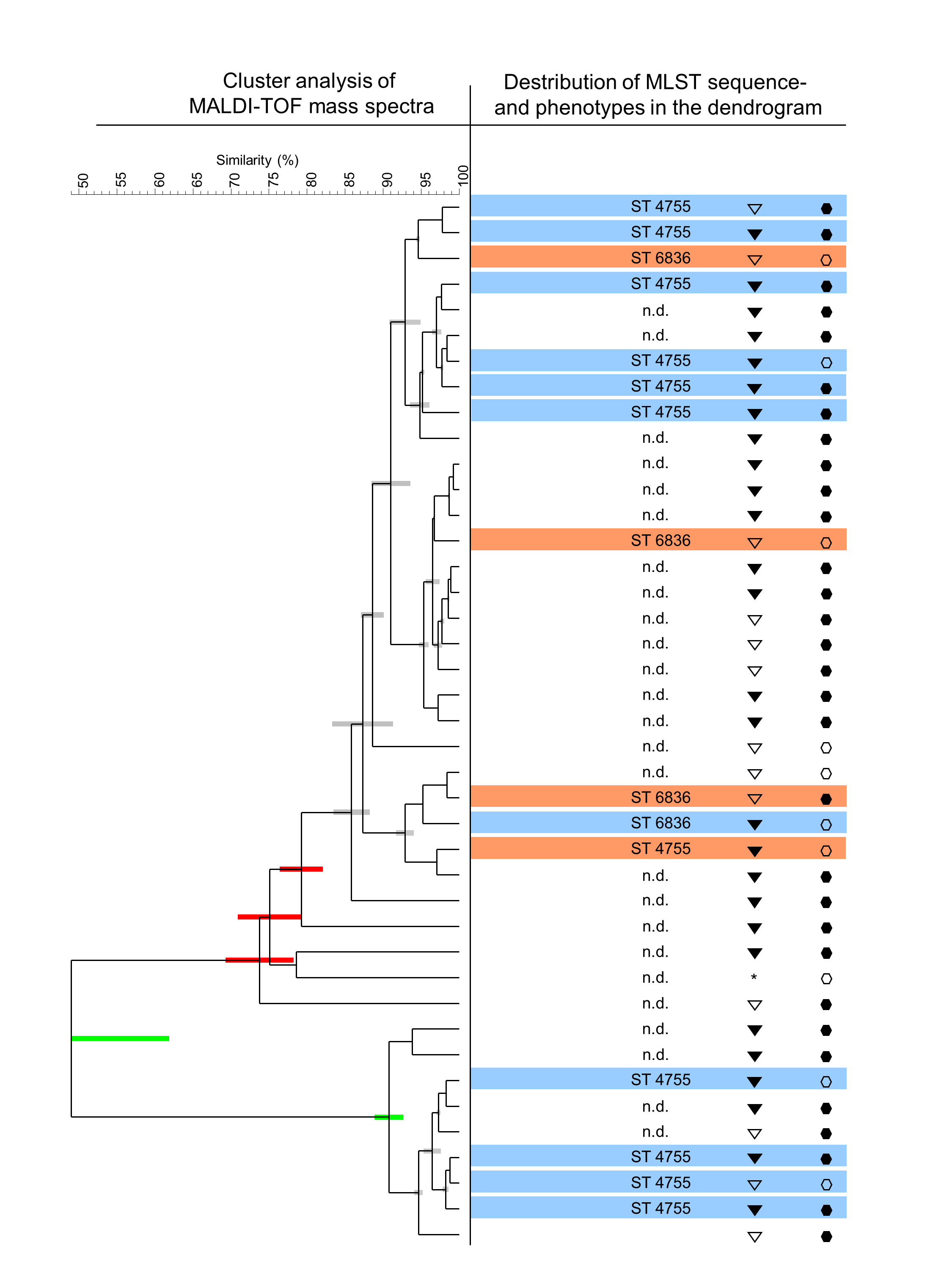

Supplement: Figure S1 — Dendrogram of MALDI-TOF mass spectra and MLST sequence and phenotypes of Campylobacter jejuni isolates. Sequence and phenotypes of isolates are not consistent with clustering of mass spectra. The dendrogram was generated using peak based pearsson correlation and UPGMA algorithm in BioNumerics 7.1. Error flags indicate branch quality by standard deviation associated with each cluster. Error flags which don't overlap indicate consistent clustering (green error flags). While overlapping error flags indicate non-consistent clusters (red error flags). Triangles display GGT activity (white triangle: no GGT activity, black triangle: GGT activity). Hexagons display phage susceptibility (white hexagon: non susceptible, black hexagon: susceptible). * Test result with low sensitivity: no distinct GGT phenotype. n.d. ST of this isolate was not tested. (TIF) [file pone.0094782.s001.tif]
